# Guanethidine Enhances the Antibacterial Activity of Rifampicin Against Multidrug-Resistant Bacteria

**DOI:** 10.3390/microorganisms12112207

**Published:** 2024-10-31

**Authors:** Xiaoou Zhao, Zhendu Zhang, Lizai Liu, Duojia Wang, Xin Zhang, Luobing Zhao, Yunhui Zhao, Xiangshu Jin, Lei Wang, Xiaoxiao Liu

**Affiliations:** Institute of Animal Husbandry and Veterinary Medicine, Jilin Academy of Agricultural Science, Kemao Street No.186, Gongzhuling 136100, China; zhaoxo4466@163.com (X.Z.); zhangzhendu@126.com (Z.Z.); 15662165476@163.com (L.L.); 15104487977@163.com (D.W.); 16624441717@163.com (X.Z.); a13630969537@163.com (L.Z.); yunhui0328@126.com (Y.Z.); 13894469000@163.com (X.J.)

**Keywords:** antibiotic adjuvant, guanethidine, rifampicin, proton motive force, antibiotic resistance

## Abstract

The escalating global threat of antibiotic resistance necessitates innovative strategies, such as the combination of antibiotics with adjuvants. Monotherapy with rifampicin is more likely to induce resistance in pathogens compared to other antibiotics. Herein, we found that the antihypertensive drug guanethidine enhanced the activity of rifampicin against certain clinically resistant Gram-negative bacteria, resulting in a reduction of up to 128-fold in the minimum inhibitory concentration. In infected animal models, this combination has achieved treatment benefits, including increased survival and decreased bacterial burden. The antimicrobial mechanism of guanethidine in synergy with rifampicin involves the disruption of the outer membrane of Gram-negative bacteria, leading to dissipation of the proton motive force. This results in an increase in reactive oxygen species and a reduction in ATP synthesis, severely disturbing energy metabolism and ultimately increasing bacterial mortality. In summary, guanethidine has the potential to become a novel adjuvant for rifampicin, offering a new option for the treatment of clinical Gram-negative bacterial infections.

## 1. Introduction

The rapid escalation of antibiotic resistance coupled with the scarcity of new antibiotics has resulted in drug-resistant bacterial infections emerging as a severe global threat, with projections estimating up to 10 million deaths by 2050 [1,2,3]. In the face of multidrug-resistant (MDR) bacterial infections, particularly those involving Gram-negative bacteria, monotherapy with antibiotics may no longer suffice [4]. These bacteria are characterised by a unique double-layered cell membrane structure, particularly the presence of an outer membrane [5]. Even polymyxins, including polymyxin B and colistin, which are considered the last line of defence against MDR Gram-negative infections, are witnessing increasing resistance in clinical isolates [6,7,8]. The pipeline for new antibiotic development has largely dried up since the 1980s, leading to a critical lack of effective treatment options for resistant Gram-negative bacterial infections [9,10]. The combination of antibiotics with adjuvants has emerged as a novel therapeutic approach, offering a more accessible alternative in clinical treatment [11,12,13,14]. This strategy has successfully mitigated resistance to β-lactam antibiotics (piperacillin/tazobactam, amoxicillin/clavulanic acid) and has been widely applied in clinical practice [15,16,17]. Furthermore, reports suggest a synergistic effect when combining antibiotics with non-antibiotic agents (doxycycline/metformin, carbapenems/auranofin) against multidrug-resistant Gram-negative bacteria [18,19,20,21].

Rifampicin is an effective broad-spectrum antibiotic whose primary mode of action involves inhibiting the transcription process of various bacteria and viruses by blocking RNA polymerase. It is utilized in the treatment and prevention of diseases caused by a range of microorganisms, including *Mycobacterium tuberculosis*, *Escherichia coli*, *Salmonella*, and *Mycoplasma* [22,23,24]. However, a critical consideration for rifampicin is its relatively high rate of resistance development. Compared to other antibiotics, the monotherapy use of rifampicin is more likely to promote resistance in pathogens. The main mechanisms of resistance to rifampicin include target modification, reduced drug uptake, overexpression of drug efflux pumps, and bacterial biofilm formation [25,26]. A classic example is the combination of rifampicin and isoniazid, which is one of the main treatment strategies for tuberculosis, with the aim of increasing therapeutic efficacy and delaying the development of drug resistance [23,27]. Consequently, rifampicin is better suited as part of a combination therapy regimen to mitigate the emergence of resistance. In this scenario, the application of antibiotic adjuvants may become an effective strategy to enhance the efficacy of rifampicin and reduce the risk of resistance development. Famotidine, for instance, can be used as an adjuvant for Rifampicin [28].

In this study, we found that the antihypertensive drug guanethidine synergised with rifampicin to enhance antimicrobial activity against multidrug-resistant Gram-negative bacteria [29], reducing the minimum inhibitory concentration (MIC) of rifampicin by up to 128-fold, thereby enhancing its therapeutic effect. Notably, guanethidine itself does not possess direct antibacterial activity (MIC ≥ 5 mg/mL). Further study, we found that guanethidine could disrupt the permeability of the outer membrane of Gram-negative bacteria, thereby interfering with the proton motive force, leading to the dissipation of the membrane potential and a compensatory increase in the pH gradient. These disrupted the electron transport chain in bacteria, resulting in increased accumulation of reactive oxygen species and reduced ATP synthesis, disrupting bacterial energy metabolism and thereby increasing bacterial mortality. Guanethidine improved the survival rate of infected animal models and significantly reduced the bacterial load in major organs. Additionally, from a chemical perspective, guanethidine and rifampicin are two independent molecules that produce a synergistic effect rather than functioning by forming a complex together.

## 2. Result

### 2.1. Guanethidine Synergistically Enhances the Antimicrobial Efficacy of Rifampicin Against Multidrug-Resistant Gram-Negative Bacteria

**Figure 1:**
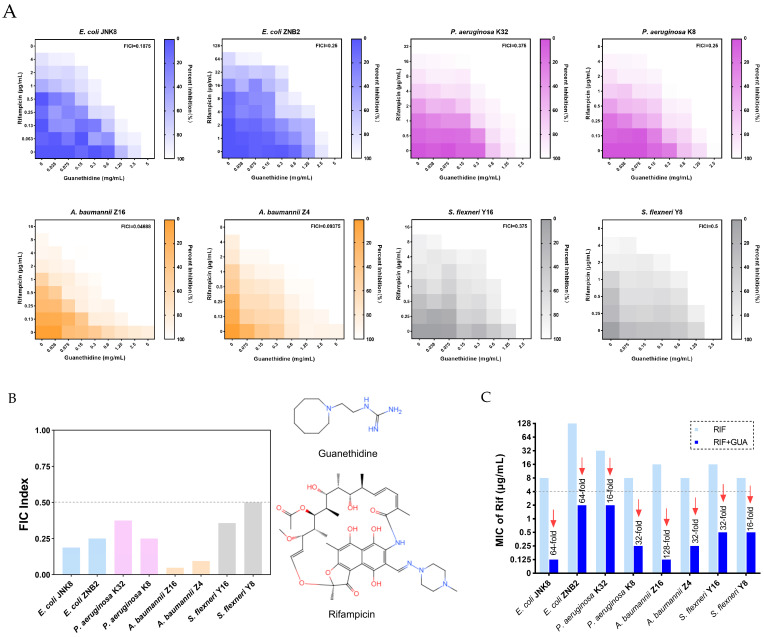
Antibacterial activity of guanethidine in combination with rifampicin against the bacteria. (**A**) The chequerboard method yielded results for the combination of guanethidine and rifampicin against *E. coli* JNB2, *E. coli* ZNB2, *P. aeruginosa* K32, *P. aeruginosa* K8, *A. baumannii* Z16, *A. baumannii* Z4, *S. flexneri* Y16, and *S. flexneri* Y8, with optical density at 600 nm (OD600) measured as a proxy for bacterial growth. The maximum values of the horizontal and vertical coordinates indicate the MICs of antibiotic adjuvants and antimicrobial drugs against the tested bacteria, respectively. Synergy is defined as a FICI of ≤0.5. All experiments were performed using biological replicates. (**B**) The FICI values guanethidine and rifampicin for *E. coli* JNB2, *E. coli* ZNB2, *P. aeruginosa* K32, *P. aeruginosa* K8, *A. baumannii* Z16, *A. baumannii* Z4, *S. flexneri* Y16, and *S. flexneri* Y8. (**C**) MICs of rifampicin with or without guanethidine (2.5 mg/mL), the fold reduction in MIC values. According to the CLSI 2020 and SFM, the sensitivity to rifampicin of a strain is restored when its MIC is ≤4 μg/mL. All experiments were performed with 2 biological replicates [31,32].

To assess the therapeutic potential of combining guanethidine with rifampicin against resistant Gram-negative bacteria, the fractional inhibitory concentration index (FICI) is used as an evaluation metric. A FICI value of ≤0.5 signifies a synergistic effect between the two substances [30]. The combination of guanethidine and rifampicin exerts a synergistic antibacterial effect on certain multidrug-resistant strains, including *Escherichia coli*, *Pseudomonas aeruginosa*, *Acinetobacter baumannii*, and *Shigella flexneri* (all FICI values are below 0.5) (Figure 1A,B). The antibiotic resistance pattern of these strains is shown in Table 1. When combined with guanethidine at a concentration of 1/2MIC (and 1/16MIC for *A. baumannii*), the MIC of rifampicin is reduced by 16 to 128-fold, thereby significantly enhancing antibacterial efficacy. Moreover, guanethidine is effective in restoring the susceptibility of resistant bacteria to rifampicin, with the MIC for all strains decreasing to below 4 μg/mL (Figure 1C).

**Figure 2:**
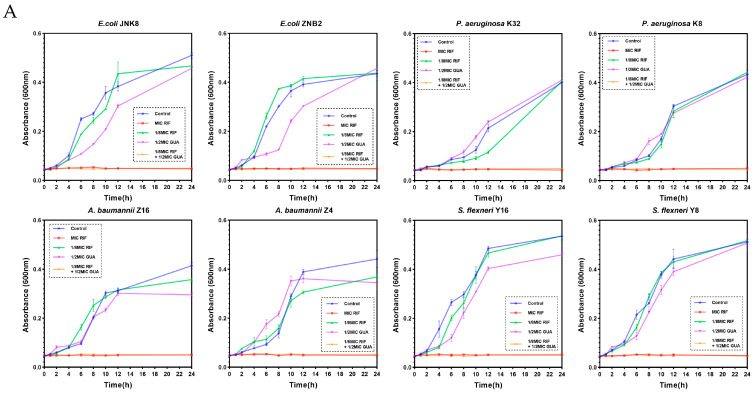

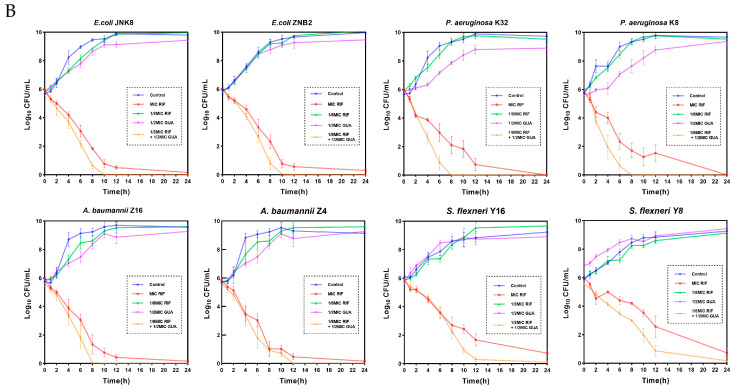
Antibacterial efficacy of guanethidine in combination with rifampicin. (**A**) Growth curves of guanethidine combined with rifampicin within 24 h. (**B**) Time-survival curves of guanethidine combined with rifampicin within 24 h. All experiments were performed with 3 biological replicates.

To further elucidate the antibacterial efficacy of the combination of guanethidine with rifampicin, we monitored bacterial growth and assessed bactericidal activity dynamics. Within 24 h, guanethidine was able to assist rifampicin in inhibiting bacterial growth and achieving complete eradication, surpassing the outcomes of either drug when used alone (Figure 2A,B).

**Figure 3:**
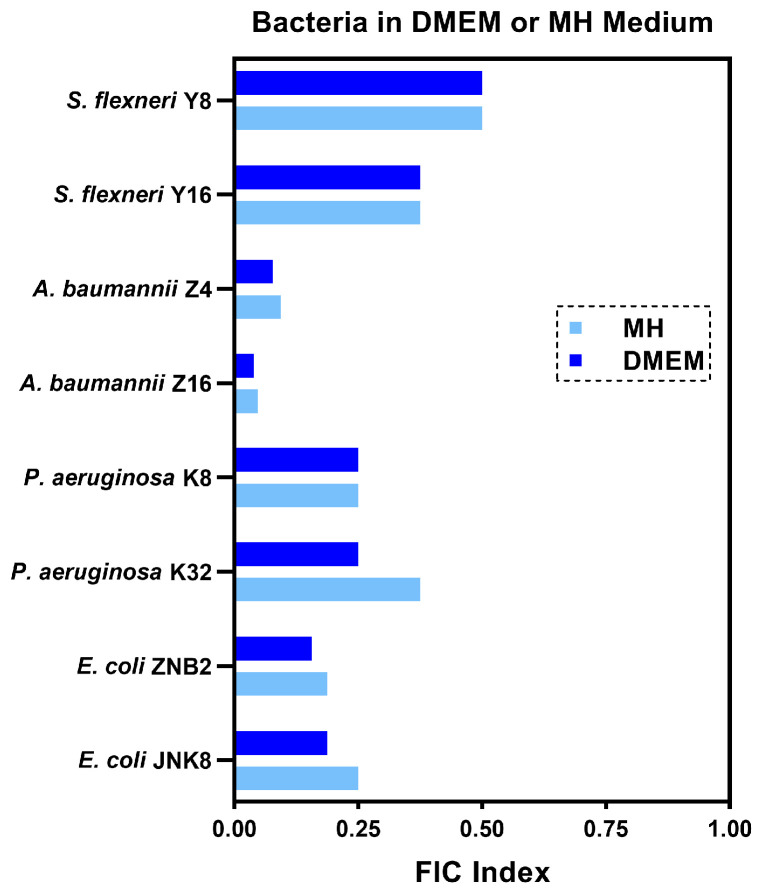
FICI values for the combination of guanethidine and rifampicin in different culture media (MH or DMEM). Synergy is defined as a FICI of ≤0.5. All experiments were performed with 2 biological replicates.

To simulate better the in vivo physiological conditions of animals, we chose DMEM medium supplemented with 1% serum to evaluate the synergistic effect. We found that the FICI values for the eight resistant strains were similar to those obtained in MH medium, indicating that guanethidine can still enhance the activity of rifampicin against these bacteria under simulated physiological conditions (Figure 3).

### 2.2. Guanethidine Affects the Integrity of Bacterial Cell Membranes

**Figure 4:**
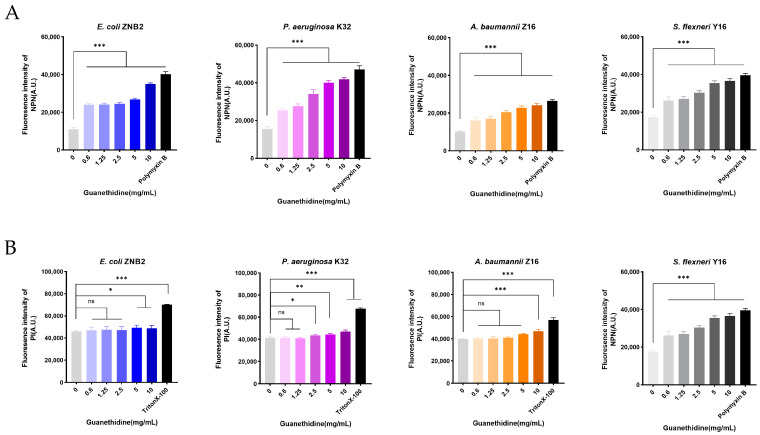
Effect of guanethidine on cell membranes. (**A**) Impact of different guanethidine concentrations on the outer membrane, assessed by NPN fluorescence intensity. (**B**) Impact on the inner membrane, assessed by PI fluorescence intensity. The significance of differences was analysed by one-way ANOVA, ns, *p* > 0.05; *, *p* < 0.05; **, *p* < 0.01; ***, *p* < 0.001. All experiments were performed with 5 biological replicates.

The bacterial cell membrane is an important target for the antimicrobial mechanisms [33,34]. We utilized a hydrophobic fluorescent probe 1-N-phenylnaphthaleneamine (NPN) to assess guanethidine’s effect on the integrity of the outer membrane (OM), while propidium iodide (PI), another fluorescent probe, was employed to evaluate its impact on the inner membrane (IM). In order to simplify the experimental procedure, we selected four strains from the eight bacterial species for further experimental research. In comparison with the blank control group, the NPN fluorescence intensity showed a significant dose-dependent enhancement. In this test, polymyxin B, a commonly used bacterial outer membrane disruptor, was chosen as a positive control to verify the reliability and sensitivity of the experimental system. (Figure 4A), However, only at high concentrations of guanethidine was there a significant increase in PI fluorescence intensity compared to the blank control. Triton X-100, which acts as a cell membrane lysing agent and is capable of disrupting the bacterial inner membrane, was used as a positive control in this text to establish a standard response for the text. (Figure 4B). In brief, guanethidine can affect the integrity of the outer membrane, with significant impact on the integrity of the inner membrane observed only at high concentrations.

### 2.3. Mg^2+^ Affects the Synergistic Effect of Guanethidine and Rifampicin

**Figure 5:**
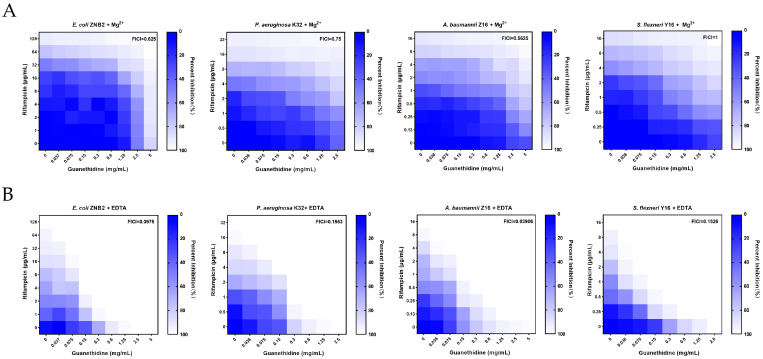
Influence of Mg^2+^ on the synergistic effect of guanethidine and rifampicin. (**A**) Chequerboard assay with the addition of Mg^2+^. (**B**) Chequerboard assay with the addition of EDTA. Synergism is defined as FICI ≤ 0.5. All experiments were performed with 2 biological replicates.

Magnesium ions (Mg^2+^) play a pivotal role in the bacterial OM by binding to lipopolysaccharides, thereby enhancing the stability of the OM and aiding in resistance against destructive external factors such as antibiotics and detergents [35,36]. Moreover, Mg^2+^ regulates the permeability of the OM, influencing the absorption of nutrients and the elimination of waste products [37]. Here, the addition of Mg^2+^ results in an increase in the FICI value, indicating a weakening of the synergistic effect (Figure 5A), while the addition of the metal chelating agent EDTA results in a decrease in the FICI value, indicating an enhancement of the synergistic effect (Figure 5B). In brief, Mg^2+^ abrogates the potentiating effect of guanethidine on rifampicin, while EDTA strengthens their synergistic action. This is consistent with Mg^2+^ strengthening OM structure and EDTA inhibiting this process.

### 2.4. Guanethidine Affects PMF

**Figure 6:**
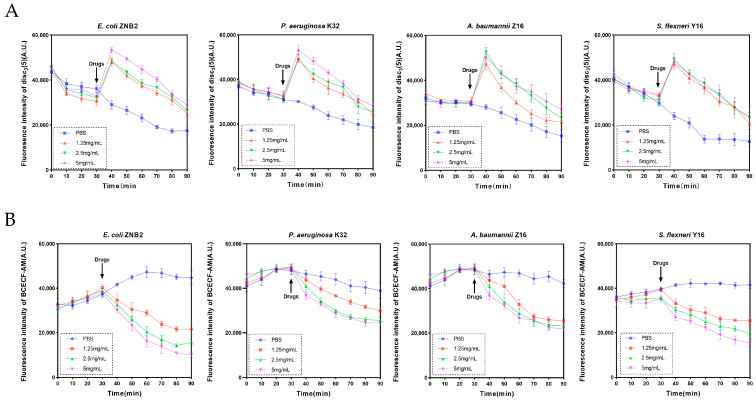
Effect of guanethidine on PMF. (**A**) The effect of guanethidine on ΔΨ was evaluated by monitoring the fluorescence intensity of disc_3_(5) over time. (**B**) The effect of guanethidine on ΔpH was assessed by measuring the fluorescence intensity of BCECF-AM. Significance of differences was analysed by one-way ANOVA, ns, *p* > 0.05; *, *p* < 0.05; **, *p* < 0.01; ***, *p* < 0.001. All experiments were performed with biological replicates. All experiments were performed with 5 biological replicates.

Disruption of membrane integrity disturbs the bacterial homeostasis, leading to internal metabolic disorders such as the dissipation of the proton motive force (PMF) [38]. Bacterial proton motive force refers to an energy form produced by the difference in proton concentration and charge on both sides of the bacterial cell membrane, which is composed of electrochemical gradient (ΔΨ) and proton concentration gradient (ΔpH) [39,40]. To monitor the changes in PMF, we utilised two fluorescent probes: disc_3_(5) for detecting alterations in the transmembrane ΔΨ, and BCECF-AM for monitoring changes in ΔpH. After the fluorescence stabilised for 30 min, different concentrations of guanethidine were added, and the fluorescence intensity was measured. When guanidine is added, the fluorescence intensity of dis_3_(5) initially rises rapidly and then gradually decreases, which is associated with the disturbance of the cell membrane potential (Figure 6A). Concurrently, the fluorescence intensity of BCECF-AM, a pH-sensitive probe, progressively decreases, indicating a decline in intracellular pH, which is a compensatory response to maintain the stability of the intracellular environment (Figure 6B). In conclusion, guanethidine can alter the membrane potential of bacteria, leading to compensatory regulation of the pH gradient, thereby affecting the proton motive force of bacteria.

### 2.5. Guanethidine Leads to Increased Intracellular ROS Accumulation and Reduced ATP Levels in Bacteria

**Figure 7:**
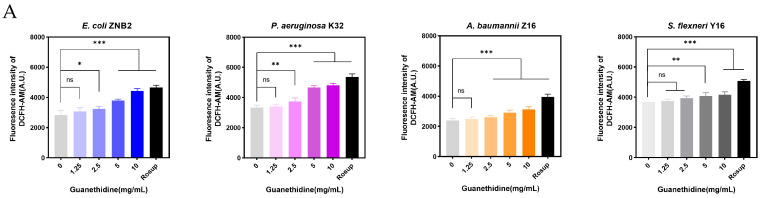

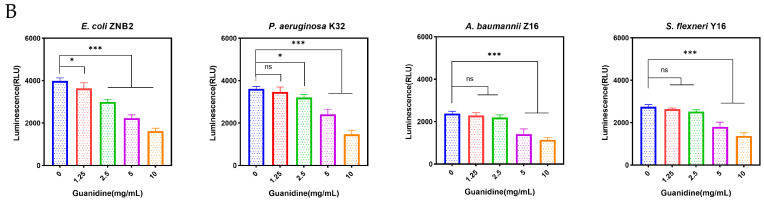
Effects of guanethidine on intracellular ROS accumulation and ATP levels in bacteria. (**A**) The effect of different concentrations of guanethidine on the accumulation of intracellular reactive oxygen species. (**B**) The effect of different concentrations of guanethidine on intracellular ATP levels. Significance of differences was analysed by one-way ANOVA, ns, *p* > 0.05; *, *p* < 0.05; **, *p* < 0.01; ***, *p* < 0.001. All experiments were performed with 3 biological replicates.

The proton motive force, as the core of bacterial energy metabolism, influences the production of reactive oxygen species (ROS) during electron transfer in the respiratory chain and is utilised by bacteria through ATP synthase to convert it into ATP [40,41]. These processes are crucial for bacterial survival. To detect ROS levels, we used the oxidative stress indicator 2,7-dichlorodihydrofluorescein diacetate (DCFH-DA), and used an ATP assay kit containing luciferase to detect ATP. As the concentration of guanethidine increased, so did the fluorescence intensity of DCFH-DA, indicating an accumulation of reactive oxygen species (ROS) (Figure 7A). Additionally, guanethidine significantly decreased luminescence intensity with increasing dosage, suggesting a significant reduction in ATP levels (Figure 7B). Guanethidine exacerbates bacterial death via ATP depletion and oxidative stress

### 2.6. The Extracellular pH Affects the Synergistic Effect of Guanethidine with Rifampicin

**Figure 8:**
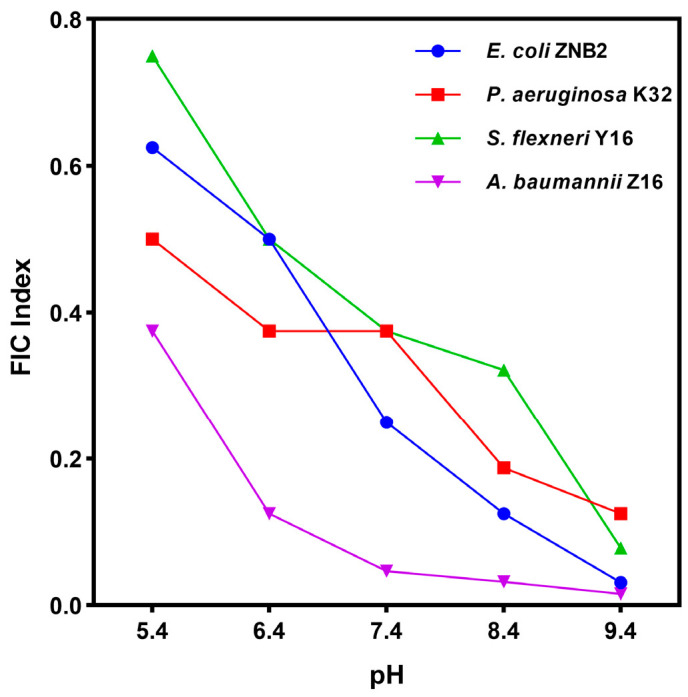
FICI of rifampicin combined with guanethidine is correlated with extracellular pH. All experiments were performed with 2 biological replicates.

Altering the extracellular pH of bacteria directly affects their proton motive force (PMF), which is determined by the proton concentration gradient (ΔpH) across the cell membrane [42]. We found that as the extracellular pH increased (becoming more alkaline), the FICI value decreased, and there was a stronger synergistic effect between guanethidine and rifampicin. However, when the extracellular pH decreased (becoming more acidic), the FICI value increased, resulting in a weaker synergistic effect (Figure 8). The guanidinium molecule contains guanidinium groups, which are susceptible to protonation by accepting protons (in which the nitrogen atom in the guanidinium group is easily protonated) and is therefore a strong base for guanidinium. In alkaline environments, the protonation of guanethidine led to an increase in proton flow across the bacterial cell membrane, exacerbating the dissipation of the proton motive force potential (PMF) and thus promoting bacterial death. However, in the acidic environment, the effect of protonation of guanethidine on the change in pH (ΔpH) was diminished due to the higher proton concentration, resulting in a less significant antimicrobial synergistic effect than in the alkaline environment. In addition, the positive charge generated by guanethidine protonation was able to interact with the negative charge on the surface of the bacterial cell membrane (mainly from the phosphate groups in the phospholipid molecules and the carboxyl groups of some proteins) via electrostatic attraction, enhancing the affinity of guanethidine for the bacterial membrane. This property further explains why the synergistic antimicrobial effect of guanethidine and rifampicin is more pronounced in alkaline environments and relatively weaker in acidic environments.

### 2.7. Synergistic Action of Guanethidine and Rifampicin as Independent Molecules Without Complex Formation

**Figure 9:**
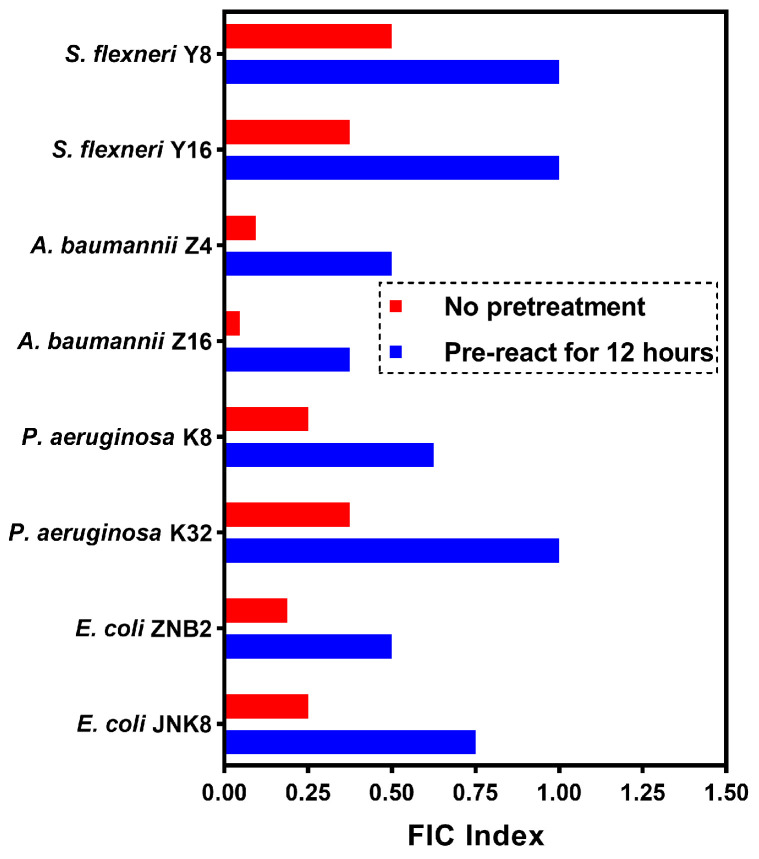
The FICI value of the rifampicin–guanethidine combination pre-reacted for 12 h was compared with the FICI value of the same combination without pre-reaction. All experiments were performed with 2 biological replicates.

Guanethidine, as a strongly basic compound, may undergo a salt-forming reaction with the acidic phenolic hydroxyl group of the bipolar compound rifampicin [23,43]. We found that when guanidine and rifampicin were mixed and pre-reacted for 12 h before the addition of the bacterial suspension to be tested, the FICI value increased compared to the negative control group, indicating that the synergistic antibacterial efficacy of the two was weakened (Figure 9). Thus, it appears that the formation of a salt complex between guanethidine and rifampicin does not enhance their bactericidal efficacy; instead, it may reduce the capability of guanidine to exert its synergistic effect. From a chemical structure perspective, guanethidine is a small-molecule compound that increases in water solubility after forming a salt. Rifampicin, on the other hand, is a large-molecule lipid-soluble compound. Gram-negative bacteria have an outer membrane that protects them, and the pathways by which guanethidine and rifampicin enter the bacterial cell interior are different. Therefore, they exert a synergistic antibacterial effect through their respective independent mechanisms.

### 2.8. The Combination of Guanethidine and Rifampicin Increased Survival and Decreased Bacterial Burden in Infected Animals

**Figure 10:**
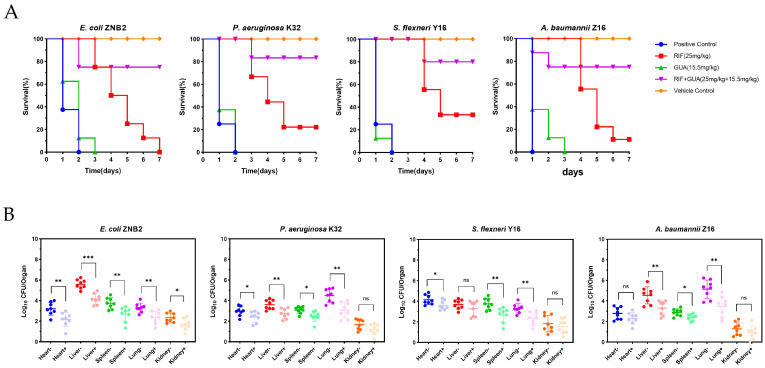
Efficacy of guanethidine combined with rifampicin in vivo. (**A**) Effect of guanethidine combined with rifampicin on survival of infected mice. (**B**) Comparison of bacterial loads in the heart, liver, spleen, and lungs of infected mice treated with the combination of guanethidine and rifampicin versus rifampicin alone. *t*-test, ns, *p* > 0.05; *, *p* < 0.05; **, *p* < 0.01; ***, *p* < 0.001.

The ideal antibiotic adjuvants should demonstrate strong in vitro synergy and robust in vivo therapeutic effects, thereby improving the survival duration and rate of infected animals [28,44]. Compared with rifampicin alone, the combination of guanethidine and rifampicin significantly increased the 7-day survival rate of infected model animals (Figure 10A). Additionally, in critical organs like the heart, liver, spleen, lungs, and kidneys, the bacterial load in model animals treated with the combination of guanethidine and rifampicin was significantly reduced compared to those treated with rifampicin alone. Notably, the most substantial decrease in bacterial load was observed in the heart, lungs, and kidneys. (Figure 10B).

## 3. Discussion

The resistance of bacteria to antibiotics has become one of the most severe global threats to human health, and the COVID-19 pandemic has further exacerbated the situation [45,46]. Gram-negative pathogens are more difficult to treat compared to Gram-positive pathogens due to their highly impermeable outer membrane, which acts as a barrier to many antibiotics [47,48,49]. The emergence of resistance to polymyxins, considered the last line of defence, has further intensified this challenge [50]. The combination of medications may or will become an effective strategy to address this challenging issue [12,51].

Guanidine compounds are a compelling chemical class characterised by their ability to become protonated in physiological environments, thereby carrying a positive charge [52,53]. This property enables guanidine groups to form hydrogen bonds or electrostatic interactions with potential bacterial targets, thereby occupying a significant role in the design and discovery of antibiotics [52]. Streptomycin, an antibiotic containing a guanidine group, is a typical representative of this class of compounds [54]. Furthermore, the introduction of guanidine-functionalised groups into polycarbonates can serve as an adjuvant, significantly enhancing the antimicrobial efficacy of various antibiotics [53]. These findings highlight the immense potential of guanidine compounds in the development of novel antimicrobial adjuvants. Guanethidine, a structurally simple guanidine compound, is clinically used primarily for the treatment of hypertension and does not possess antimicrobial activity [43]. In this study, we discovered a synergistic effect between guanethidine and rifampicin, which can enhance the antibacterial activity of rifampicin against multidrug-resistant Gram-negative bacteria.

Rifampicin is a lipid-soluble, high-molecular-weight antibiotic that exerts its antibacterial activity only upon entering bacterial cells. However, the outer membrane of Gram-negative bacteria makes it difficult for rifampicin to penetrate into the cells [55]. Adjuvants can help rifampicin overcome this challenge. Guanidinium-functionalised polycarbonates (such as ptE-20) can reverse rifampicin resistance phenotypes and enhance antimicrobial efficacy by membrane translocation [56,57]. The short linear antimicrobial peptide SLAP-L25 can disrupt cell membrane permeability, thereby increasing the efficacy of vancomycin, rifampicin, and colistin E against multidrug-resistant *Escherichia coli* [58]. Similar to these findings, we found that guanethidine can also compromise the integrity of the outer membrane of certain Gram-negative bacteria, as indicated by changes in fluorescence intensity between the inner and outer membranes and the observed synergistic effects of Mg^2+^ on this process. This disruption dissipates the PMF and impairs electron transfer in the respiratory chain, leading to an increased accumulation of ROS, reduced ATP synthesis, and a cascade of reactions that severely disturb bacterial energy metabolism, ultimately increasing bacterial mortality. The mechanism is analogous to that of lopatadine (an adjuvant for minocycline) or metformin (an adjuvant for doxycycline) [18,59]. The changes in fluorescence intensity of the fluorescent probes for ΔpH and ΔΨ confirm these results. Moreover, in vitro pH changes significantly affect the synergistic effect of guanethidine and rifampicin, further substantiating this conclusion. Interestingly, the antihypertensive mechanism of action of guanethidine, as an antihypertensive drug, mainly acts on the nerve endings and vascular system and does not directly affect the integrity of cell membranes, so the antihypertensive effect of guanethidine is not directly related to the disruption of cell membranes.

In addition, the death of Gram-negative bacteria is associated with cell membrane changes, such as antimicrobial peptides [60]. In the present study, we found that guanethidine, although affecting bacterial cell membrane completeness, did not possess direct antibacterial activity (minimum inhibitory concentration MIC ≥ 5 mg/mL). This may be due to the amphiphilic character of the antimicrobial peptide as well as the diversity of spatial structures. In contrast, guanethidine, as a small-molecule compound, has a relatively simple structure. Although most antimicrobial peptides and guanethidine contain a guanidine group, the core structure of guanethidine contains only one guanidine group, which does not allow it to directly exert complex antimicrobial effects as antimicrobial peptides do. However, guanethidine is able to synergise with rifampicin to enhance its antimicrobial efficacy.

Guanethidine is a small molecule with strong alkalinity due to the guanidine group, which significantly improves its water solubility after salt formation. Rifampicin, on the other hand, is an amphoteric lipophilic macromolecule. Considering the presence of an outer membrane in Gram-negative bacteria, guanethidine and rifampicin enter the bacterial cell interior through different pathways. Given the guanidine group in guanethidine and the multiple hydroxyl groups in the rifampicin molecule, a chemical reaction between the two may occur. To determine whether they work independently or form complexes, we first incubated guanethidine and rifampicin together in the same environment for 12 h and then added a bacterial suspension to assess their synergistic effects by measuring FICI. We found that the synergistic effect was reduced in the pre-reaction group compared to the control group that did not pre-react, especially with *Pseudomonas aeruginosa* and *Shigella flexneri*, in which the synergistic effect was no longer present. We speculate that guanethidine and rifampicin may achieve synergistic antibacterial effects through their distinct but potentially complementary mechanisms of action.

An ideal adjuvant for antibiotics should significantly enhance the antibacterial activity of antibiotics in vitro and effectively improve the survival time and likelihood of survival in infected animal models [28]. In this study, compared with the use of rifampicin alone, the combined use of guanethidine and rifampicin significantly increased the survival rate of infected animal models and reduced the number of bacteria in major organs. This effect is similar to that of another rifampicin adjuvant, famotidine. In summary, guanethidine has the potential to be used as an adjuvant for rifampicin, especially in the fight against Gram-negative bacteria that have developed resistance to traditional antibiotics.

## 4. Materials and Methods

### 4.1. Bacteria and Reagents

The strains involved in this study were grown in Mueller-Hinton broth (MHB, Qingdao Hopebio, Qingdao, China) or Dulbecco’s Modified Eagle’s Medium (DMEM, Beijing Baiteke Biotechnology, Beijing, China). The drugs and reagents were procured from Macklin (Shanghai, China). The kits were purchased from Beyotime (Shanghai, China).

### 4.2. MIC Measurements

The MICs of all compounds were determined using the standard broth microdilution method according to Clinical and Laboratory Standards Institute 2020 (CLSI 2020) [31]. Single colonies were selected, inoculated into Mueller-Hinton broth (MHB), and then incubated on a shaker at 37 °C (200 rpm) until a logarithmic growth phase was reached and the bacterial concentration was adjusted to 10^6^ CFU/mL. The drug was diluted 2-fold in a 96-well microtitre plate and then topped up with an equal volume of the 10^6^ CFU/mL bacterial solution. Incubation was performed at 37 °C for 16 to 24 h. The MIC was determined as the lowest concentration of antibiotic at which there was no visible bacterial growth.

### 4.3. FIC Index Determination

Chequerboard assays were employed to assess the combined effects of compounds and antibiotics using fractional inhibitory concentration (FIC) indexes [61,62]. Antibacterial drugs were diluted by a factor of 2 along the *x*-axis, while compounds were similarly diluted along the *y*-axis. Each well was filled with a mixture containing 10^6^ CFU/mL bacterial solution and then incubated at 37 °C for 16 to 24 h. The optical density of each well at 600 nm was measured using a Multiskan FC Microplate reader (Thermo, America).

The FIC index (FICI) was determined by using the following formula:

FIC index = MIC_ab_/MIC_a_ + MIC_ba_/MIC_b_ = FIC_a_ + FIC_b_

where MIC_a_ is the MIC of compound A alone, MIC_ab_ is the MIC of compound A in combination with compound B, MIC_b_ is the MIC of compound B alone; MIC_ba_ is the MIC of compound B in combination with compound A, FIC_a_ is the FIC index of compound A, and FIC_b_ is the FIC index of compound B. Synergistic effect is defined as an FIC index of ≤ 0.5, additive effect is defined as an FIC index of >0.5 and ≤1.0, no effect is defined as an FIC index of ≥1.0 and <4.0, and an antagonistic effect is defined as an FIC index of ≥4.0. Additionally, supplementary reagents or medications may be included as needed (e.g., 10 mM Mg^2+^ and 10 mM EDTA), or the pH of the culture medium can be adjusted accordingly.

### 4.4. Determination of Growth Curve and Time-Kill Curve

Determination of growth curves was carried out as follows. The bacterial suspension with a 0.5 McFarland turbidity was diluted 100-fold and then exposed to different drug concentrations for incubation, with absorbance levels being measured at various time intervals using a microplate reader. The group without any drug treatment served as the baseline control group.

Determination of kill curve was carried out as follows. The bacterial liquid prepared in the previous step was consistently thinned out for the purpose of counting colonies. 100 μL of the diluted sample was spread on MH agar plates and incubated at 37 °C for 24 h. The number of colonies on each plate was counted and multiplied by the corresponding dilution [28,63]. The group without any drug treatment served as the baseline control group.

### 4.5. Fluorescence Assay

In the fluorescence assay, all bacterial agents used for measurement were treated similarly [18]. In brief, after overnight incubation of the strains at 37 °C and 200 rpm, they were washed three times using HEPES II (5 mM) or PBS (10 mM) buffer. The bacterial suspensions were adjusted to an OD600 of 0.5, followed by the addition of a fluorescent dye incubated at 37 °C for 30 min. Bacteria labelled with the fluorescent dye were exposed to different concentrations of the drug and then analysed at specific excitation and emission wavelengths using a fluorescent microplate reader (Molecular Devices, San Jose, CA, USA).

#### 4.5.1. Outer Membrane Permeability Assay

HEPES washing solution and NPN (10 μM) fluorescence probe were employed [18]. The fluorescence of the culture content was measured using an excitation wavelength of 350 nm and an emission wavelength of 420 nm. The negative control group was not treated with guanethidine, while the positive control group was treated with 10 mM polymyxin B as a control.

#### 4.5.2. Inner Membrane Permeability Assay

PBS wash solution and a PI (10 nM) fluorescent probe were employed [63]. The fluorescence of the cultured content was assessed using an excitation wavelength at 535 nm and an emission wavelength at 615 nm. The negative control group was not treated with guanethidine. The positive control used for comparison was 1% Triton X-100.

#### 4.5.3. Membrane Potential Gradient Assay

Bacteria in the exponential phase were washed three times with 5 mM HEPES II buffer, followed by the addition of 0.5 mM disc_3_(5) fluorescent dye. The bacteria were then co-incubated with various drug concentrations, and their fluorescence intensity was measured at an excitation wavelength of 622 nm and emission wavelengths of 670 nm [62]. The PBS treatment group served as the reference control group.

#### 4.5.4. pH Gradient Assay

Bacteria in the exponential phase were washed three times with 5 mM HEPES II buffer, followed by the addition of 20 μM BCECF-AM fluorescent dye. The bacteria were then co-incubated with various drug concentrations, and their fluorescence intensity was measured at an excitation wavelength of 488 nm and emission wavelengths of 522 nm [18]. The PBS treatment group served as the reference control group.

#### 4.5.5. Total ROS Measurement

Bacteria in the exponential phase were washed three times with 5 mM HEPES II buffer, followed by the addition of 10 μM DCFH-DA fluorescent dye. The bacteria were then co-incubated with various drug concentrations, and their fluorescence intensity was measured at an excitation wavelength of 485 nm and emission wavelengths of 535 nm [18]. The negative control group was not treated with guanethidine, while the positive control group was treated with 10 µM Rosup as a control.

### 4.6. ATP Determination

The ATP levels inside the cells were measured using an ATP Assay Kit (Beyotime, Shanghai, China) [18]. Cells in the exponential growth phase were washed with PBS three times. After adding different concentrations of drugs and incubating for 1 h, they were then centrifuged at a low temperature. The resulting supernatant was mixed with the ATP working solution, allowed to stand for 5 min, and its absorbance was measured at 636 nm. Subsequently, the obtained value was used in a formula to determine the concentration of ATP.

### 4.7. Mouse Intraperitoneal Infection Model

Mice were randomly divided into 4 components, each group of 8, male and female half, and fed for 7 days in a closed environment to adapt to the environment [28]. Mice were intraperitoneally injected with test strains suspension (bacterial solution concentration 10^8^ CFU/mL), and drugs were intraperitoneally injected an hour later. The mice were injected with guanethidine (15.5 mg/kg), rifampicin (25 mg/kg), guanethidine (15.5 mg/kg), and rifampicin (25 mg/kg) in the abdomen. The non-model group, treated only with solvent, served as the negative control. The positive control was injected with the same amount of carrier solvent after model construction, and the survival of the mice within 7 days was observed and recorded. The laboratory animal use licence number is JNK20230901-1.

### 4.8. Measurement of Bacterial Load in the Organs of the Infected Mouse Model

The mice were randomly divided into 2 groups, 8 mice in each group, half male and half female, and were kept under closed conditions for 7 days to adapt to the environment. Mice were intraperitoneally injected with the test strains suspension (the concentration of bacterial solution was 10^6^ CFU/mL). Furthermore, 1 h later, the mice were intraperitoneally injected with rifampicin (25 mg/kg), and the other groups were injected with guanethidine (15.5 mg/kg) and rifampicin (25 mg/kg). After 24 h of infection, the heart, liver, spleen, lungs, and kidneys were dissected under sterile conditions. The tissues were homogenised and serially diluted, followed by spreading onto MacConkey agar culture medium containing doxycycline resistance. The plates were then incubated at 37 °C for 20 h, and the bacterial colonies were counted.

### 4.9. Statistical Analysis

Statistical analysis was performed using an unpaired *t*-test between two groups and a one-way ANOVA among multiple groups. SPSS22.0 was used for statistical analysis. Quantitative data were expressed as mean ± standard deviation; *p*-values were considered statistically significant (* *p* <0.05, ** *p* < 0.01, and *** *p* < 0.001; ns represents insignificant).

## 5. Conclusions

In this study, we have revealed an innovative drug combination strategy by combining guanethidine, an antihypertensive drug, with rifampicin to significantly enhance the latter’s antibacterial activity against multidrug-resistant Gram-negative bacteria such as *Escherichia coli*, *Pseudomonas aeruginosa*, *Acinetobacter baumannii*, and *Shigella flexneri*. Guanethidine affects the permeability of the bacterial outer membrane, leading to dissipation of the membrane potential and subsequently resulting in an increased compensatory pH gradient. This leads to the dissipation of the proton motive force, which in turn disrupts the electron transport chain. This disruption contributes to an increase in reactive oxygen species accumulation, a reduction in ATP synthesis, and interference with bacterial energy metabolism, ultimately resulting in an elevated bacterial mortality rate. Concurrently, this combination can effectively lower the MIC of rifampicin, improve the survival rate in infected animal models, and reduce the bacterial load. This discovery provides a new approach for treating multidrug-resistant bacterial infections and has potential clinical application value. Therefore, guanethidine is expected to be a novel adjuvant of rifampicin, providing a new therapeutic option for the clinical treatment of multidrug-resistant Gram-negative bacterial infections.

## Figures and Tables

**Table 1:** The antibiotic patterns of the strains in this study.

Strains	Source	Antibiotics (MIC μg/mL)									
		Rifampicin	Tetracycline	Ampicillin	Vancomycin	Colistin	Enrofloxacin	Azithromycin	Florfenicol	Gentamicin	Sulfamonomethoxine Sodium
*E. coli* JNB2	Pig	8/R	64/R	256/R	16/R	1/S	8/R	0.5/S	16/R	4/I	256/R
*E. coli* ZNB2	Faeces	128/R	128/R	128/R	16/R	8/R	1/S	1/S	128/R	2/S	256/R
*P. aeruginosa* K32	Chicken	32/R	8/R	128/R	8/R	1/S	2/I	0.5/S	4/I	4/I	64/R
*P. aeruginosa* K8	Water	8/R	16/R	128/R	8/R	0.5/S	2/I	1/S	4/I	8/R	128/R
*A. baumannii* Z16	Chicken	16/R	128/R	128/R	8/R	0.5/S	4/I	0.5/S	16/R	4/I	256/R
*A. baumannii* Z4	Dog	8/R	32/R	128/R	8/R	1/S	2/I	0.5/S	4/I	16/R	64/R
*S. flexneri* Y16	Water	16/R	256/R	256/R	16/R	4/R	1/S	1/S	32/R	4/I	128/R
*S. flexneri* Y8	Faeces	8/R	128/R	128/R	8/R	0.5/S	1/S	0.5/S	2/S	4/I	128/R

According to CLSI 2020 and SFM, R is for resistant, I is for intermediate between resistant and sensitive, and S is for sensitive.

## Data Availability

Data are contained within the article.
